# Health Informatics: Engaging Modern Healthcare Units: A Brief Overview

**DOI:** 10.3389/fpubh.2022.854688

**Published:** 2022-04-29

**Authors:** M. J. Yogesh, J. Karthikeyan

**Affiliations:** School of Information Technology and Engineering, Vellore Institute of Technology, Vellore, India

**Keywords:** health informatics, public health, information systems, health policy, public health systems

## Abstract

In the current scenario, with a large amount of unstructured data, Health Informatics is gaining traction, allowing Healthcare Units to leverage and make meaningful insights for doctors and decision-makers with relevant information to scale operations and predict the future view of treatments *via* Information Systems Communication. Now, around the world, massive amounts of data are being collected and analyzed for better patient diagnosis and treatment, improving public health systems and assisting government agencies in designing and implementing public health policies, instilling confidence in future generations who want to use better public health systems. This article provides an overview of the HL7 FHIR Architecture, including the workflow state, linkages, and various informatics approaches used in healthcare units. The article discusses future trends and directions in Health Informatics for successful application to provide public health safety. With the advancement of technology, healthcare units face new issues that must be addressed with appropriate adoption policies and standards.

## 1. Introduction

Machine Learning is the fastest-growing topic in computer science today, and Health Informatics (HI) is the most difficult problem to solve (1, 2).

Emerging economies are increasing their investments in healthcare, which makes sense and encourages health professionals to adopt sound frameworks and regulatory standards, as well as health IT, to improve the quality and efficacy of care (3). In this expanding field, new age occupations can be established. This new field has the potential to be a lucrative career path in the future. With a clear flow of information across many medical subsystems, adoption of electronic health record systems (EHRs) will improve the health care system going forward (4).

Big Data is frequently employed in the field of health informatics, as new data is constantly pouring into the system, requiring analysis and interpretation in order to make rational decisions (5, 6).

A bigger impact can be accomplished in healthcare has ushered in a new era for healthcare companies to improve decision-making through the comprehensive integration of data from a range of sources, allowing for much faster and more effective decision-making (7). Within and outside of the medical business, computational health informatics is an emerging study field (7–9).

In recent years, the healthcare industry has seen a rapid growth in medical and healthcare data, which can be used to improve facilities and public health care utilization and implementation by modern healthcare units using novel treatment and diagnosis methodologies, which gives citizens confidence in using the best public healthcare services available and aids governments in developing better healthcare policies (10).

Computerized systems for analysis and diagnosis were first adopted by health professionals. More recent technology are making it easier for people to make better decisions (11, 12).

Health Informatics' promise to improve public health activities would be fully realized if information science and technology were applied (13).

In a complex social and economic environment, the issue is thus how to increase the quality of offered healthcare services while lowering prices. Information and communication technologies (ICT) have been shown to help healthcare systems increase productivity, which has resulted in significant cost savings in operations and service delivery. For administrative and healthcare objectives, ICTs have already proven to be quite effective. New prospects for new medical equipment and systems are opening up as ICTs become smaller, quicker, wireless, and remotely controlled.

The Internet and the web have recently brought up new possibilities for increasing the response time of health-care services while also lowering costs. It is clear that we are in the early stages of a new era that will fundamentally alter the way healthcare services are provided. This will help us acquire the public's trust in using high-quality healthcare services.

New e-Health services and technology should be researched, developed, promoted, and disseminated with significant effort. With the present pandemic (COVID-19) sweeping the globe, increasing ICT use has demonstrated that healthcare will become more contactless in the future, with fresh means of treating patients and providing healthcare services emerging. This is a popular yet difficult research subject since it necessitates interdisciplinary competence (14).

The major purpose of Health Informatics is to increase our understanding of medicine and medical practice by using real-world medical data. In the subject of healthcare, health informatics is a blend of information science and computer science (15).

Big data in healthcare is intimidating not only because to its sheer magnitude, but also due to the variety of data types and the pace with which it must be managed. To gain people' trust and give quality healthcare services, all health service providers are now putting in extra effort to use the most up-to-date technologies to provide health services and advanced treatments.

Various requirements drive innovation in this industry, such as finding appropriate accommodation with standardization and coordinating the acquisition and implementation of newer healthcare systems and services on a national/international level.

With the present COVID-19 scenario, investments in this sector are gaining steam with new age healthcare units in many nations, and growing economies such as India and China will play a vital part in providing good and quality healthcare services in the future.

As a result, New Age Healthcare Units and Systems will play a critical role in dramatically lowering costs, making Public Healthcare Systems more dependable and instilling citizens' confidence in using inexpensive healthcare.

## 2. Related Work

Big data is a term used to describe a significant volume of data that is collected and stored today and has outgrown standard data management and analysis solutions. Solutions like Hadoop and Spark, according to Roger Fyre and Mark McKenney, have arisen to solve some of the big data concerns (16).

Researchers have used Hadoop to implement a variety of parallel processing algorithms to efficiently handle geographical data (17, 18). Multistage map and reduce algorithms, which generate on-demand indexes and retain persistent indexes, are examples of these techniques (19).

Much of the current work on predictive analytics, particularly in clinical contexts, is aimed at improving health and financial outcomes, which will aid in making better decisions (20). Data mining, which is defined as the processing and modeling of huge amounts of medical/health data to identify previously unknown patterns or associations, is one of the most important machine learning approaches (21, 22).

Data collection for diseases such as cancer and neurological disorders in order to improve disease prognosis (23, 24). Cancer detection and diagnosis, as well as other health-related issues, have been made possible because to these breakthroughs. Here, prominent research in the topic of health monitoring and informatics is discussed, which can be used to verify future research (25).

Machine Learning is crucial in the testing and development of various models that take into account clinical and other important medical characteristics for decision-making.Medical imaging, which incorporates capabilities such as image segmentation, image registration, annotation, and database retrieval, is one of the most famous examples of newer medical technologies that can be utilized for decision making in the future. For all of this, updated ML/DL models for speedier decision-making can be constructed (26).

Machine learning/data science researchers are in high demand for developing algorithms that adapt to changing data. Deep Learning (DL) is now being used to solve more difficult problems in the healthcare/informatics arena (26, 27).

Holzinger et al. (28) discussed many approaches to developing an explainable model for the medical domain were examined. Prediction explanations can be useful in a variety of situations, including teaching, learning, research, and even court. The demand for interpretable and explainable models is growing in the medical field. They need to be able to re-enact the decision-making and knowledge extraction processes. In their article, Ribeiro et al. (29) discussed how machine learning models are black boxes. Understanding the reasons for predictions can help to build trust. It can be used to assess model performance and construct better, more accurate, and correct models by providing insights into the model. Ribeiro et al. (29) propose the LIME algorithm for explaining predictions of any model. This article by Bahdanau et al. (30) deals with neural machine translation, but the model proposed can be used in a variety of other applications.

## 3. Introduction to HL7 FHIR Architecture

In the last two decades, electronic health records (EHR) have been widely implemented in the United States to improve health-care quality, increase patient happiness, and reduce health-care costs (31–33). As growing countries such as India, China, and Bangladesh experiment with innovative ways to establish EHR systems, it will significantly aid in the development of effective public health systems.

FHIR's basic idea was to create a set of resources and then create HTTP-based REST application programming interfaces (APIs) to access and use these resources. FHIR uses components called resources to access and perform operations on patient's health data at the granular level. This feature distinguishes FHIR from all other standards because it was not present in any earlier version of HL7 (v2, v3) or the HL7 clinical document architecture (CDA).

The fundamental building blocks of FHIR are the so-called resources, which are generic definitions of common health care categories (for example, patient, observation, practitioner, device, condition). For data interchange and resource serialization, FHIR employs JavaScript object syntax and XML structures. FHIR not only supports RESTful resource exchange but also manages and documents an interoperability paradigm.

FHIR has grown in popularity and is being increasingly used by the health care industry since its inception. In 2018, six major technology companies, including Microsoft, IBM, Amazon, and Google, vowed to remove barriers to health care interoperability and signed a statement mentioning FHIR as an emerging standard for the interchange of health data. With incorporation of Substitutable Medical Applications Reusable Technologies (SMART), a platform for inter operable applications (34), FHIR can be expected to attract even more attraction in digital health in the future.

The use of FHIR for medical data transmission has the potential to deliver benefits in a wide range of disciplines, including mobile health apps, electronic health records (EHRs), precision medicine, wearable devices, big data analytics, and clinical decision support.

**Figure 1:**
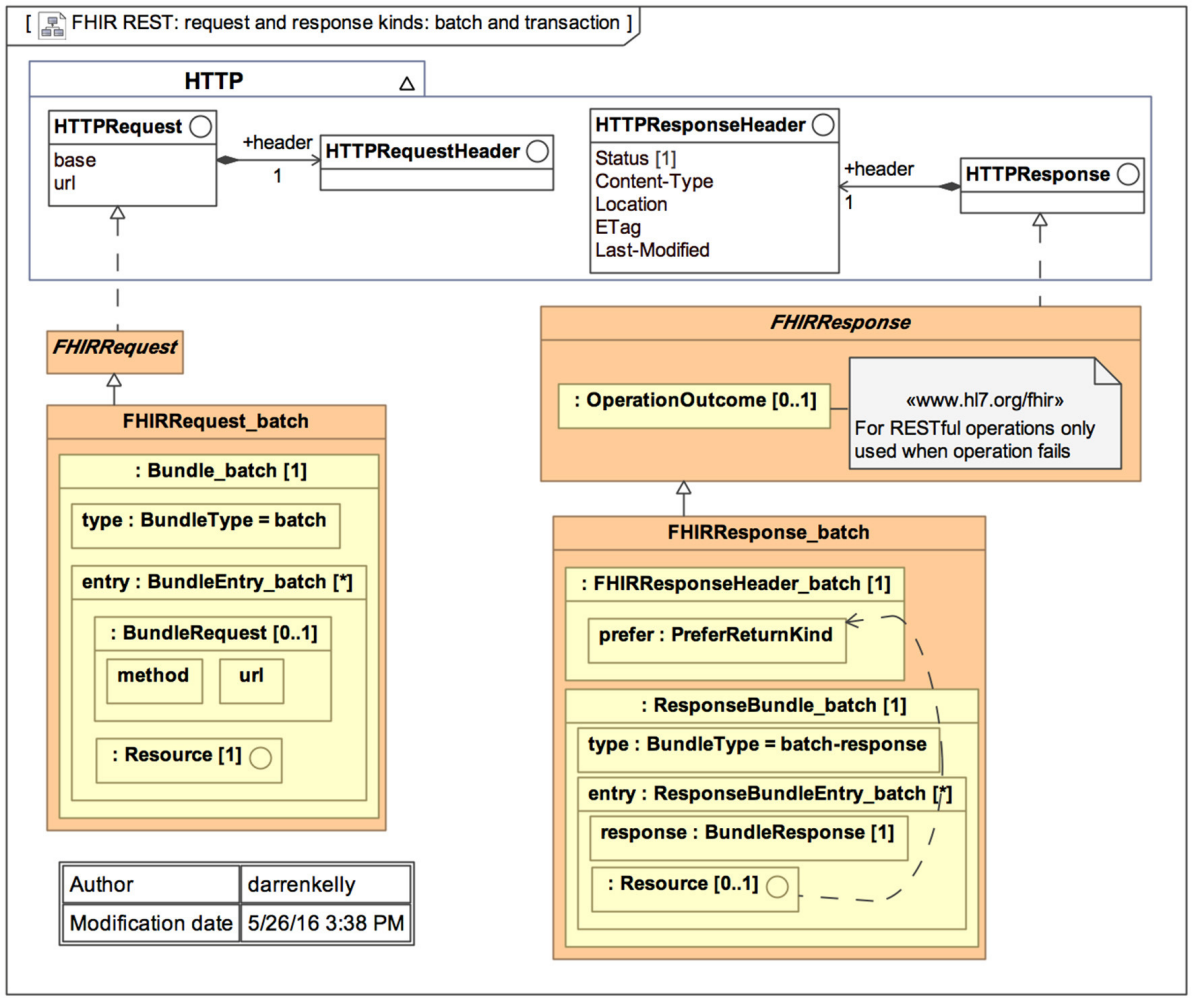
General architecture of the Fast Health Interoperability Resources (FHIR) standard (35).

The primary goal of FHIR is to reduce implementation complexity while maintaining information integrity. Furthermore, this new standard integrates the benefits of existing HL7 standards (v2, v3, and CDA) and is projected to overcome their drawbacks. FHIR enables developers to create standardized browser applications that allow users to access clinical data from any health care system, regardless of the operating systems and devices used. Figure 1 represents the general architecture of FHIR (35).

### 3.1. FHIR for Patient Access to Medical Records

FHIR, or Fast Healthcare Interoperability Resources, is an HL7 standard for electronically transferring healthcare information. The CMS Interoperability and Patient Access final regulation, announced in 2020, mandates all CMS-regulated payers to use FHIR version 4. Unlike earlier releases, the fourth iteration is backward compatible, ensuring that software suppliers' solutions will not become obsolete when a new FHIR version is released.

The FHIR (pronounced “fire”) standard defines a collection of HTTP-based RESTful APIs that allow healthcare platforms to exchange and share data in XML or JSON format. FHIR offers mobile apps, which users can obtain from the Apple App Store or Google Play in order to access their medical records and claims data.

FHIR's basic exchangeable data piece is known as a resource. Each resource is formatted similarly and contains roughly the same amount of data. It offers information about patient demographics, diagnosis, prescriptions, allergies, care plans, family history, claims, and so on, depending on the kind. They span the complete healthcare workflow and can be used independently or as part of a larger document.

**Figure 2:**
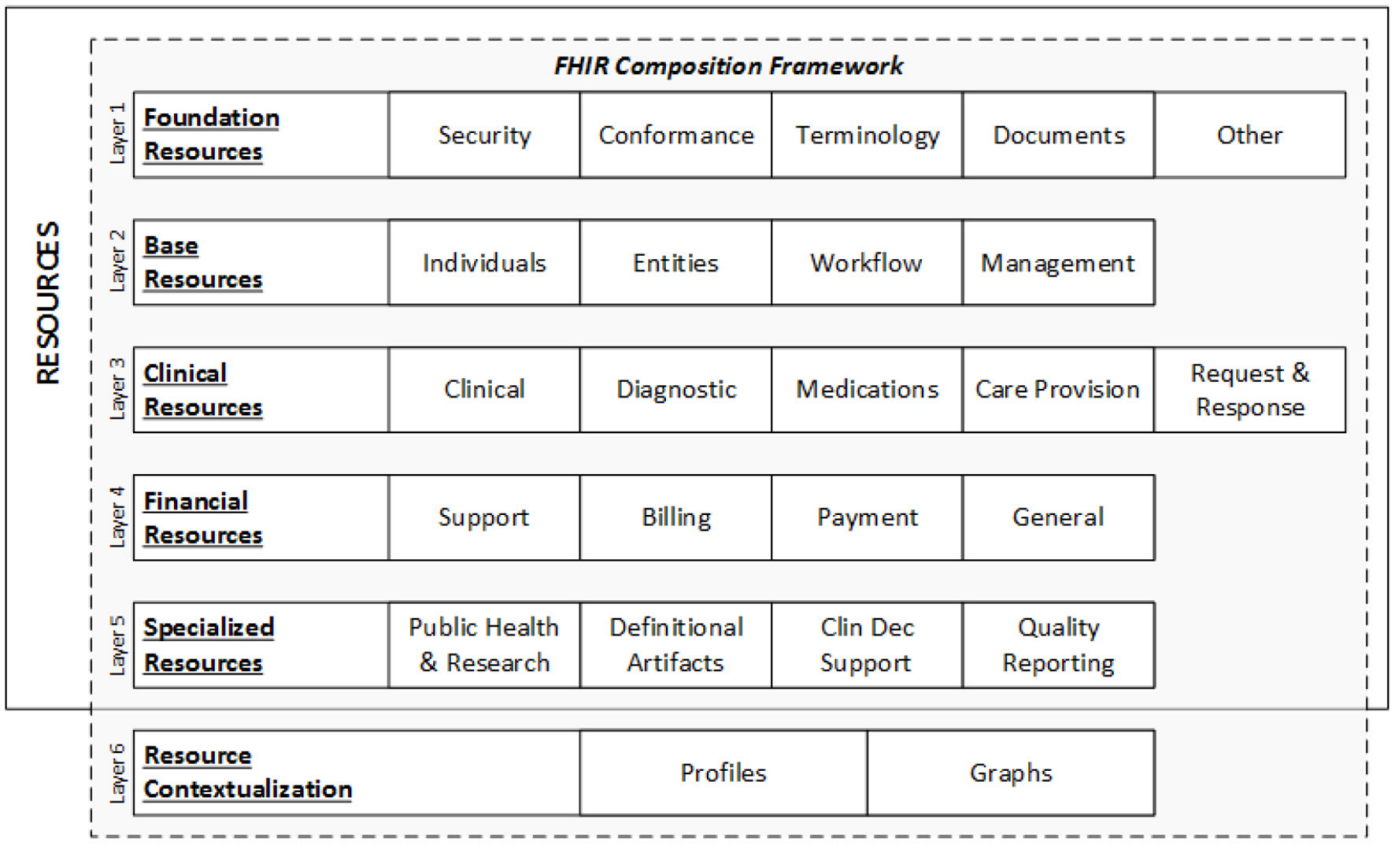
FHIR data layers and resources (35).

Each resource is given a unique ID, and many parties—health systems, insurers, patients, or software developers—can access the underlying data element using an API. Figure 2 represents the data layers and resources of FHIR (35).

### 3.2. FHIR Resource

A resource is the smallest discrete concept that can be independently maintained and is the lowest feasible unit of a transaction (36). As a result, a resource is a known identity that provides useful data. Each resource has distinct bounds and differs from all others. A resource should be provided in sufficient depth to specify and enable the process's medical data interchange. The FHIR community has specified over 150 resources till date, according to the most recent FHIR version (R4) (37).

There are five key categories in which these resources can be found: (1) Administrative: location, organization, device, patient, and group; (2) Clinical: CarePan, diagnostics, medication, allergy, and family history; (3) Financial: billing, payment, and support; (4) Infrastructure: conformance, document, and message profile; and (5) Workflow: encounter, scheduling, and order.

FHIR is fast gaining popularity due to its dynamic properties. FHIR is projected to quickly become a symbol for clinical data interchange in the health-care industry.

### 3.3. Workflow Description

Workflow is a critical component of healthcare; orders, care regimens, and referrals drive the majority of activity in in-patient settings, as well as a significant amount of activity in community care. FHIR (Fast Health Interoperability Resources) is concerned with workflow when it is necessary to share information about workflow state or relationships, when it is necessary to coordinate or drive the execution of workflow across systems, and when it is necessary to specify permissible actions, dependencies, and behavior requirements.

### 3.4. Workflow State and Relationships

FHIR does not have to be used for workflow execution. Orders, care plans, test findings, hospital admissions, claim payments, and other documents can all be exchanged utilizing FHIR resources without the need for an FHIR transaction to solicit fulfillment of those orders or request payment of those claims. Because it necessitates a greater level of standardization, inter-operable support for workflow execution is a more advanced FHIR activity. Interoperable workflow execution necessitates the standardization of processes, roles, and activities across multiple systems, rather than just the data to be exchanged.

Even if FHIR is not used for workflow execution, there is still a requirement to standardize workflow data elements: how does an event or a result point to the order that allowed it? How are parent and child steps tied together? How does a care plan know which protocol it is following?

**Figure 3:**
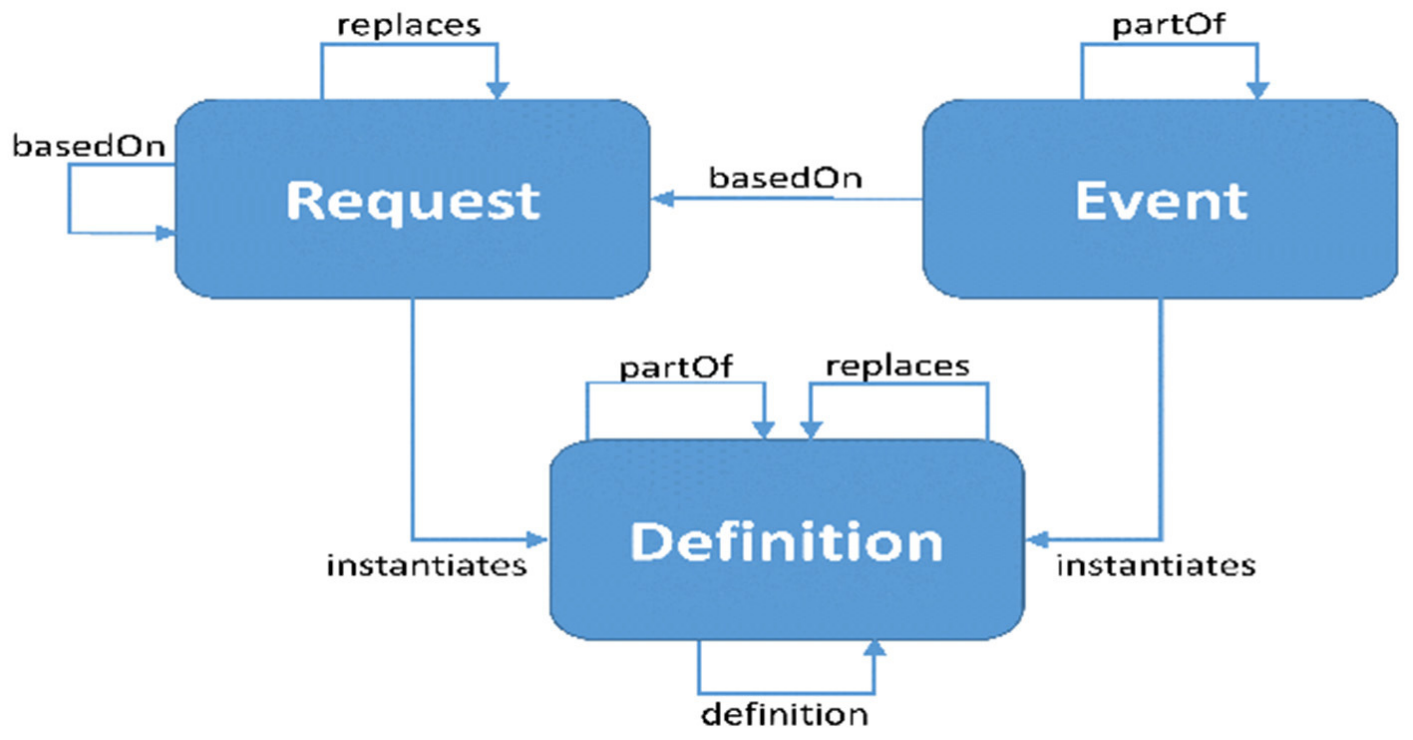
Work flow relations Fast Health Interoperability Resources (FHIR) standard (38).

FHIR distinguishes three types of resources engaged in activities: requests, events, and definitions. Each of these categories is associated with a “pattern.” Resources in that category are encouraged to follow their specific pattern. These patterns provide conventional elements that are common to the majority of resources in each category. Work groups are anticipated to align with common domain behavior and requirements as more authoritative than “desired” architectural patterns, therefore strict conformance is not necessary. When a pattern capability is assessed to be “not common, but nonetheless relevant” for a given resource, it may be supplied through extensions rather than core parts. Figure 3 represents the work flow relations of FHIR Standard (38).

## 4. Overview of Health Informatics

Health informatics involves more than merely automating routine tasks. With contemporary technology developments in machine learning and deep learning, it is possible to redesign systems using methodologies that were previously impossible or not even considered (26).

Such a study is computationally expensive and can now be handled by the latest IBM POWER9 processors with GPU capabilities, which was previously impossible because the data was not available in electronic form and the number of possible symptoms/incident patterns was too big to manage. Early detection of patterns that can assist anticipate what kind of treatment or diagnosis can be offered to such patients has improved dramatically (39).

In the future, modern healthcare units will make use of such a framework for successful treatment delivery for society as a whole, making effective use of data obtained from such systems by extracting insights that assist decision makers such as doctors, hospital owners, and health policymakers.

In terms of vital signs, an example of classification in healthcare informatics is clearly handled (40, 41). The prediction of a patient's breathing rate obtained from sensors is an example of regression (42). In the future, Bayesian Inference will be used to make better predictions in the domain of health informatics.

Some of the most pressing future difficulties in healthcare informatics will be addressed by developing systems based on the principles listed below, which are not commonly employed in this sector:

- Multi-task Learning
  Traditional machine learning frameworks consider only one learner attempting to solve a single task. However, in many applications, there are multiple tasks that label the same data instances differently. When the tasks are related, the information learned from each task can be used to improve learning of other tasks. Learning relevant tasks concurrently, rather than learning each task independently, is thus advantageous. Multi-task learning makes use of the intrinsic relationships between multiple tasks to improve generalization performance. It benefits all tasks by leveraging task relatedness and shared information across relevant tasks (43).
- Transfer Learning
  Traditional machine learning technology has had a lot of success and has been used in a lot of practical applications, but it still has certain limits in some real-world settings. Machine learning works best when there are a lot of labeled training cases with the same distribution as the test data. In many cases, however, gathering sufficient training data is costly, time-consuming, or even impossible. Semi-supervised learning can help to alleviate this difficulty by removing the requirement for large amounts of labeled data. A semisupervised approach typically requires a small amount of labeled data and a large amount of unlabeled data to improve learning accuracy. However, in many cases, unlabeled instances are difficult to collect, making the resulting traditional models unsatisfactory (44).
- Multi-agent-Hybrid Systems Multi-agent systems are networks of interconnected autonomous agents in which the behavior of neighboring agents influences the dynamics of each agent.Because of the increasing importance of multi-agent systems, there is a growing interest in coordination control to ensure consensus, flocking, containment, formation, rendezvous, and so on. To better understand multi-agent coordination, a variety of dynamic models of agents have been developed over the last two decades. Furthermore, many mathematical methods are used in the analysis and control of multi-agent systems. For more information, see survey article (45) and the references therein (46).
- Representation Learning
  Patient-specific data such as vital signs, medications, laboratory measures, observations, clinical notes, fluid balance, procedure codes, diagnostic codes, and so on are all included in modern EHR systems. Clinicians originally employed the codes and their hierarchies, as well as their associated ontologies, for internal administrative and invoicing functions. Recent deep learning algorithms, on the other hand, have attempted to project discrete codes into vector space, identify intrinsic commonalities between medical concepts, more accurately depict patients' health, and perform more precise predicting tasks. Word embedding and unsupervised learning have been used to examine medical concepts and patient representations in general (47).

Health informatics has a number of long-term benefits in terms of research and healthcare delivery that can be used to create a sustainable ecosystem. ICTs aid in the enrichment of relevant data for analysis and decision-making by health professionals. Following the pandemic (COVID-19), new age healthcare units will arise, with increased investment and research spending making public healthcare more accessible. As a result, solutions are required to manage the massive amounts of data created by medical equipment and healthcare systems, allowing for effective storage and retrieval in real-time data analysis and decision-making.

### 4.1. Informatics Approaches

To make electronic health data more easily usable for research, recent publications have identified the need for effective adoption and use of standards, essential data and research services, clear and consistent policies regarding data access and use, and transparent and effective governance structures (48, 49).

To achieve data quality criteria, electronic health data utilized in research frequently require standardized ontologies, additional contextual information, field transformations, and missing or contradictory data to be handled (50). For research-related data or functions, such as cohort identification and repeated extracts of source data over time, system development is frequently required (49). Organizations with expertise utilizing and enhancing their health IT infrastructure for research have shared their lessons learned in these areas, adding value to organizations with similar goals but less experience or resources (51).

For example, when preparing data for research use, organizations must understand the clinical context and structure of electronic health data, just as they do for other data uses such as decision support or population health. Individual researchers and data analysts can be relieved of their load by informatics support that spans research and operational usage of data. It is vital to evaluate and develop informatics tools and approaches by establishing processes that allow for coordinated governance and decisions informed by research users.

Investing in infrastructure to enable the use of electronic health data for research has also been shown to be beneficial to researchers by providing them with the necessary tools and expertise, to patients by providing clinical trial participation opportunities, to clinicians by enabling more rapid translation of research into practice, and to population health analysts by facilitating patient cohort views. In order to reduce project-specific IT costs, using health IT to assist research necessitates greater flexibility, increasing use of standards, and reusable ways for getting, preparing, and evaluating data (52).

Any use of operational data in research necessitates the establishment of a privacy and security framework, as well as data governance monitoring. Two initiatives, Informatics for Integrating Biology and the Bedside (i2b2) and Observational Health Data Sciences and Informatics (OHDSI), have developed informatics tools and approaches that allow researchers to query organizational participants and support transformation or analytics of relevant data to facilitate research (53, 54).

Specifically, i2b2 has standardized data models and distributed computational tools that enable for the anonymous identification of potential genomic study participants at the institution level. OHDSI also employs a single data model, which incorporates information such as health economics and health systems. The methodologies utilized in these programmes demonstrate the kind of functionality that may be required in health IT systems to better support research, as well as the types of concerns with the quality of electronic health data that regularly arise.

## 5. Status QUO in Health Informatics: An Indian Perspective

### 5.1. Health Care Delivery Systems

Despite having a solid telecommunication infrastructure, the existing systems are based on manual record keeping. The value of medical informatics in healthcare delivery has yet to be recognized by policymakers (55).

In countries such as India, health informatics is a new and emerging discipline. Its future prospects are very bright, thanks to the development of excellent infrastructure here. However, in order to implement a robust framework, this necessitates a multidisciplinary interaction with various stakeholders.

Information systems development could be another area where research is being conducted to improve the way data flows from various sources such as devices and medical equipment, allowing doctors and decision makers to make rational decisions on critical cases or equipment purchases in the future.

Digitizing all medical data also aids in the creation of a structure for patient-related data in a hospital that can be easily retrieved and searched. Finally, the development of some kind of electronic health record can be accomplished through the development of information systems (56).

### 5.2. Applications of Health Informatics

Health Informatics in India can become cost effective and ensure proper service delivery, which aids in beneficiary behavior change through the use of ICTs (57). We can create novel applications that can be used effectively by utilizing local talent and effective use of ICT in remote parts of India (58). Various governance issues can be addressed in the future with certain checks and balances in the data collection and analysis process (59). The following are some of the areas where health informatics can be used:

1. Epidemiological disease prediction
2. Disaster management
3. Awareness in Healthcare Processes
4. Healthcare in Remote areas
5. Electronic Health Records and its linkages with health systems
6. Health Statistics
7. Education and Training
8. Development of Decision Support Systems (DSS)
9. Public Health Research
10. Visualization tools for doctors
11. Recommendation Systems for Health Informatics
12. Precision Drug Prediction.

The potential of this emerging area has far-reaching benefits over a long period of time, and new and novel solutions can be built using various machine/deep learning models. There is a lot of work to be done in this area where we can use cutting-edge technology to aid/assist in the development of robust products and frameworks for public health policy (60–63).

### 5.3. Future Trends and Directions in Health Informatics

These are a few of the current trends in the field of Health Informatics that can be used to develop sustainable products, services, and health-related policies for effective implementation across the country and internationally. A few of them are listed below, and many more trends may emerge in the near future as a result of discussions with multiple stakeholders.

1. Data standards and Interoperability
2. Processes to transform medical/clinical data
3. Toolkits and Pipelines: Data Management
4. Standardized Reporting Methodologies
5. Appropriate Use of Informatics Expertise.

More trends may emerge in the future, taking into account the most recent technological advancements. Because health informatics is a new and emerging field, more research challenges may emerge, bringing forth newer perspectives in the future. To solve more difficult problems in this area, future researchers will prefer machine/deep learning methods/models (26). There are numerous other research directions being pursued in relation to various aspects of health care data such as quality, veracity, privacy, and timeliness. The following are some of the most notable data characteristics of healthcare data (9, 64):

1. **Complexity and Noise:** Because healthcare data is multisource and multimodal, it has a high level of complexity and noise. Furthermore, there are issues with impurity and missing values in high-volume data. It is difficult to deal with all of these issues, both in terms of scale and accuracy, despite the fact that a number of methods have been developed to improve data accuracy and usability (65). Because the quality of data dictates the quality of information, which in turn affects decision-making, it is vital to develop efficient big data cleansing ways to improve data quality in order to make effective and correct decisions (66).
2. **Heterogeneity:** Traditional healthcare data is frequently fragmented with multiple forms due to a lack of standardization. As a result, it is both reasonable and important to investigate and adopt universal data standards. However, due to the complexity of developing universal data standards, it is a difficult undertaking. Not only is healthcare data diverse, but there are numerous technical challenges to integrating that data for specific purposes (67). Even with standardized data formats, the multi modal character of data makes efficient fusion difficult (68), necessitating the development of advanced analytics that cope with vast amounts of multi modal data. The integration and synthesis of multi source and multi modal healthcare data on a larger scale would be a significant issue.
3. **Longitudinal Analysis:** Longitudinal data is the collection of repeated measurements of participant outcomes and possibly treatments or exposures (69), which means that “the outcome variable is repeatedly measured on the same individual on multiple occasions (70).” In recent decades, longitudinal data analysis, particularly statistical longitudinal data analysis, has gotten a lot of attention. Longitudinal studies are used to characterize normal growth and aging, as well as to evaluate the effectiveness of risk factors and therapies. It is extremely important in epidemiology, clinical research, and therapeutic evaluation. With big data analytic tools, it is possible to perform longitudinal care analysis across patients and diagnoses to identify the optimal care options.
4. **Scale:** Healthcare data is continuously expanding in quantity and scope (68). The fact that data volume is growing faster than processing power is a significant challenge in managing vast amounts of data. Several fundamental adjustments are occurring to handle this enormous transition (71). First, in recent years, CPU technology has increasingly turned its focus to parallel data processing within nodes and the packing of numerous sockets. Second, the shift to cloud computing allows for information sharing and the consolidation of multiple workloads into large-scale clusters. Third, the transformation of the traditional I/O subsystem from Hard Disk Drives (HDDs) to Solid-State Drives (SSDs), as well as other storage technologies, is reforming data processing system design and operation.
5. **Real Time:** The velocity of big data in health informatics reflects not only the rate of data collecting and processing, but also the timeliness of replies. There are various instances that call for a quick choice. For example, it would be immensely desirable to monitor and analyse a person's health condition in real time or near real time in order to predict potential disease. It would also be critical to raise the alert for a potential influenza outbreak by examining public health statistics. Although real-time analytic applications are still in their infancy in the big data era, they represent the strongest trend and most promising direction in health informatics' future (72).
6. **Privacy:** Data privacy is another major worry for future big data analytics in healthcare informatics (73). Although strong laws control more formalized EHR data, extra attention should be taken and standards should be enforced to regularize the use and dissemination of personal and sensitive information obtained from diverse sources. In addition to data privacy, there are a number of other challenges, like as data protection, data security, data safety, and the protection of doctors from liability resulting from manipulated data, that necessitate the use of specialized big data analytics to address these complicated constraints (73, 74).
7. **Visualization:** The visualization of healthcare data is crucial for exploratory or discovery analytics, which aim to investigate and discover elements that are hidden or encrypted in the data (75). Effective visualization tools will enable clinicians and physicians to explore data without the need for IT assistance (72).
8. **Multidisciplinary and Human-Computer Interaction:** Big data in health informatics is expected to be a multidisciplinary job requiring ongoing contributions from multiple topic experts (76). They include, but are not limited to, engineering scientists who provide basic big data infrastructure to collect, store, share, and manage big data; computer science data scientists who provide solutions for processing and analyzing high-volume, high-velocity healthcare data using a variety of data mining and machine-learning techniques; and clinicians and physicians from the medical domain who provide professional healthcare data analysis, personalized care, and make recommendations. Computer algorithms can struggle to find patterns and interpret results at times; consequently, it is a desirable feature for an advanced big data analysis system to be able to enable input from numerous human specialists, exchange of viewpoints, and collaborative exploration of outcomes. Furthermore, in the health sector, we sometimes do not have massive data: we are confronted with a small number of datasets or unusual events, where, for example, machine-learning algorithms suffer from insufficient training samples. In such circumstances, we require more than just automatic machine learning; we also require a person in the loop. In other words, interactive Machine Learning (iML) or “human in the loop” techniques can be used in health informatics when automatic machine-learning algorithms cannot handle rare occurrences on their own and a human expert is required to interact in the learning process (77).The interplay between computer algorithms and human specialists has the potential to improve the learning process.

## 6. Challenges in Health Informatics

There are numerous challenges in this area, particularly in a country like India, where a large population is denied affordable healthcare, which can serve as a starting point for developing and implementing robust health informatics applications, products, and Research and Development investments. The start-up/venture costs, people, and equipment are very high, and it is a niche sector in which many people are hesitant to venture into such a space/sector that can be leveraged making very good business sense (78).

To improve the way health services are delivered and implemented to the public, a multidisciplinary approach involving various sectors/stakeholders is required. These informatics systems can be used for a variety of purposes. Medical/healthcare data will be more structured and democratized by competent authorities.

The advancement of EHRs and web-based health monitoring systems will aid in rational decision making and policy framework implementation (11). There may be numerous bottlenecks in the development of ICT systems for Health Informatics (59, 79).

The majority of stakeholders are unconvinced about the benefits of Internet technologies in health care and are unfamiliar with how to use such new technology (80). Concerns about security and privacy may arise as robust healthcare systems are developed in the future (11).

Proper techniques are essential to ensure patient data confidentiality, and system security may become a concern when data policy standardization increases. Many new challenges can be encountered while developing novel and innovative ways to promote public health through the use of information technology (IT) and other computing technological advances such as Cloud Computing, Data Visualization, and Medical Informatics. Future research is required because the healthcare/informatics domain is still in its early stages, where more difficult problems can be solved and newer products and services can be spawned as a business venture. Deep Learning in Health Informatics has its own set of challenges (26).

Despite significant investments in information technology, patient safety and productivity have not improved. Preventable medical errors are the third leading cause of death in the United States, after heart disease and cancer, killing over 400,000 people each year. These blunders cost the United States over one trillion dollars per year. To combat this disease, the federal government has mandated that the healthcare industry transition to electronic health records (EHRs) and use these records to improve patient processes and outcomes (i.e., meaningful use). Emerging economies have to allocate and give incentives to healthcare units/organizations to promote the use of EHRs.

The following are the main gaps and challenges to an effective pandemic response in health information management and health informatics:

1. A lack of standards for information exchange between providers and PHAs (Public Health Authorities)
2. Issues with data collection and data quality, particularly in terms of completeness and timeliness
3. Governance, Public Policies, and Regulations.

The latter included a lack of procedures to support efficient data sharing, contact tracking, and data governance, as well as providers' concerns about privacy regulations, which resulted in insufficient data sharing.

Governance and public policy hurdles stem from chronic underfunding of public health infrastructure, as well as a lack of adequate investments in resources (particularly qualified employees) and facilities. A key difficulty was also recognized as a lack of international coordination. Many overlapping and interrelated legal, ethical, scientific, technical, technological, health equality, and privacy elements influenced how health information was managed or mismanaged during the COVID-19 global pandemic. Other long-standing systemic difficulties in health information management will need to be addressed in order to operationalize many of the data and information system recommendations (81).

The financial investment required to design, execute, and sustain e-health programmes is a key difficulty in health informatics, and Anderson cited a lack of financial backing and high initial expenses as hurdles to implementing ICT in health care (82). While health informaticians and information professionals may see future benefits from investments in ICTs, health professionals and managers may be skeptical, especially if they are satisfied with current methods of working and wish to maintain the status quo, and may see such initiatives as diverting financial resources away from under-resourced clinical care (83).

Resistance to the establishment of ICT systems by health professionals and managers can lead to further issues once the systems are in place, and the restricted adoption of health informatics applications has meant that their potential is not always achieved. Decision support systems, for example, may be ignored or overridden, and evidence-based information may have limited applicability for an individual patient. Clinicians make life-changing judgments or act in life-threatening situations, and if they don't comprehend the reasons behind computer-based decision support systems, they won't trust them or use them effectively (84).

This highlights the importance of not only involving physicians and healthcare professionals in the construction of systems and the interpretation of outcomes, but also of providing adequate explanation and information at the point of care for healthcare practitioners to trust the systems (84).

As previously stated, identifying the types of information that clinicians require, as well as the methods by which they access and utilize information, is critical in ensuring that developments not only meet the needs of the users, but are also perceived to be valuable, so that health professionals and other users will want to maximize their potential. Addressing healthcare professionals', patients', and the public's concerns about data security, as well as threats to patient privacy and confidentiality, will be critical in developing online access to patient records (85). Greater security measures integrated into system design will help boost system confidence, however the chance of third parties getting access to sensitive patient-identifiable data remains a danger (82).

Another challenge that can stall the creation and execution of health informatics efforts is quality. The real and perceived quality of data entered into systems and then used for health care is vital not only for assuring system use, but also for the safety and well-being of patients. If data is not input, or is not entered correctly, the buildup of missing or low quality data discourages others from using the system and creates additional suspicion and skepticism about future advancements. The importance of accurate and correct data will grow as lifelong electronic records are established (84), both prospectively as individuals are born and retrospectively using data acquired over an existing person's lifetime to date.

As previously noted, the earlier development of small-scale information systems within individual departments or hospitals resulted in system incompatibility and difficulties communicating or transferring data when larger-scale systems were later built. One approach to addressing this issue is to increase interoperability and employ known electronic record architectures in the creation of new systems. In addition, the lack of data standards in health presents additional challenges for moving and sharing data between systems (82). Attempts to address these issues include the creation of information-management standards such as Digital Imaging and Communications in Medicine (DICOM), Health Level Seven (HL7), and terminologies and coding systems [e.g., the International Classification of Diseases (ICD), Read coding, and Snomed] to standardize the ways in which medical conditions and diseases are represented in computer-based systems and to attempt to codify the natural language used by medical staff.

The International Classification of Diseases (ICD) was created to provide a standard method of classifying medical diagnoses for epidemiology and health-care purposes; initially, it only included causes of death, but more recent versions have included causes of morbidity, and it is now in its eleventh version (86). There are numerous challenges, and many new problems can be solved using cutting-edge technologies such as ML/DL and cloud computing (26, 87–92).

### 6.1. Health Data Standards Challenges and Possible Solutions to Them

Clearly, data standards are not lacking in the healthcare industry. SDOs (standard development organization) have created a plethora of them to address nearly every facet of communication between diverse health systems.

However, the simple fact that they exist and are available does not address all of the issues surrounding interoperability. We'll go over some of the more difficult standards issues, as well as potential solutions.

1. **Medical coding speed and accuracy issues:**
  The manual work required to convert diagnoses, treatments, services, treatment plans, and other concepts into medical codes is undertaken by professionally qualified individuals. Computer-assisted coding systems are now used by coders. However, the translation process's speed and precision are far from flawless. To that aim, high hopes are placed on AI-powered tools capable of identifying proper codes and recommending them for expert evaluation. Currently, such intelligent systems speed up coding, but they cannot completely replace humans and automate the entire process.
2. **Need for mapping between codes:**
  Each code in healthcare serves a specific purpose: SNOMED allows physicians to provide a thorough clinical picture of a patient being treated, whereas ICD-10 presents diagnoses quickly and CPT summarizes services. However, there are times when translation from one code system to another is required. As previously stated, SNOMED cannot be used for billing reasons and must be translated to ICD-10-CT. To overcome mapping issues, standard development groups experiment with various approaches.
3. **Lack of compatibility between old and new standards:**
  To comply with existing interoperability regulations, hospitals must make content described by USCDI available *via* FHIR-based APIs. But, let's face it, the truth is: Most EHR systems were designed with previous standards in mind. Some of them are only capable of importing and exporting HL7 v2 messages. Others rely heavily on C-CDA materials. Neither v2 nor C-CDA are compatible with granular USCDI data elements or FHIR basic interchangeable data blocks—resources. As a result, hospitals will require additional digital technologies and human resources to extract data from legacy formats and convert it into FHIR and USCDI-compliant parts.
4. **No two-way communication between patients and EHRs:**
  The FHIR standard enables patients to access health data through apps of their choice. However, because EHRs only allow read-only access to their systems, this is a one-way street. The software allows users to request information but does not allow them to modify or change it. Many industry experts believe that the next major difficulty for healthcare is a lack of two-way communication between medical apps and EHR systems. And, sooner or later, it will need the development of new data standards (93).

## 7. Conclusion

Research in this fresh domain of health informatics is vital because we need to be aware of the necessary discipline of health informatics where new discoveries can be realized effectively and *via* rigorous testing. There are no proven design blueprints for such a comprehensive infrastructure, and the goal is always shifting due to the nature of real-time data collecting from a variety of sources, such as patient/medical/equipment data. The future of health informatics is to create novel algorithms, models, and an ecosystem that is conducive to health professionals and decision makers.

Some of the advantages that will result from innovation in this new emerging area will be critical, such as:

- Case/Incident related Health Information Standards and Services
- Professionalism among Healthcare Units
- Various Innovative Industrial Processes
- Enhanced National and International Collaboration for Research
- Visualization Tools
- Novel Prediction Models (Machine Learning/Deep Learning/Reinforcement Learning)
- New Products and Services related to Healthcare/Health Informatics Domain
- Storage and Retrieval Mechanisms with Healthcare Related Data
- Curated New/Novel Datasets for Research Community.

Current cutting-edge health informatics research projects aim to discover new condition onset behaviors that are visible in physiological data streams earlier than traditional condition detection in critical care data (94). Cloud computing has attracted a lot of research attention, but only a small portion of the work done so far has addressed performance issues, and only a few of these have used a rigorous analytical approach (95–98).

Big data is also playing an important role in health informatics, where a large amount of data related to healthcare is generated, assisting and assisting doctors and decision makers in making rational decisions regarding patient treatment and diagnosis. Newer computational technologies may emerge that will improve the way healthcare is delivered and implemented in the future, thereby vastly improving the public healthcare system. Policymakers can propose tried-and-true use cases that can be transformed into a meaningful framework in which all stakeholders can deliberate and decide on the best model for improving the public health care system.

Health informatics is a new field with many stakeholders involved in the design and implementation of sustainable public health systems and policies for the benefit of society as a whole.

## Author Contributions

MY has written the manuscript with relevant information from the literature regarding the major challenges and gaps that the present Health Informatics system and public health system are undergoing. The manuscript was reviewed and inputs were given by JK to focus on Health Informatics and policy framework. All authors contributed to the article and approved the submitted version.

## Conflict of Interest

The authors declare that the research was conducted in the absence of any commercial or financial relationships that could be construed as a potential conflict of interest.

## Publisher's Note

All claims expressed in this article are solely those of the authors and do not necessarily represent those of their affiliated organizations, or those of the publisher, the editors and the reviewers. Any product that may be evaluated in this article, or claim that may be made by its manufacturer, is not guaranteed or endorsed by the publisher.
